# Atypical language organization in temporal lobe epilepsy revealed by a passive semantic paradigm

**DOI:** 10.1186/1471-2377-14-98

**Published:** 2014-05-06

**Authors:** Júlia Miró, Pablo Ripollés, Diana López-Barroso, Adrià Vilà-Balló, Montse Juncadella, Ruth de Diego-Balaguer, Josep Marco-Pallares, Antoni Rodríguez-Fornells, Mercè Falip

**Affiliations:** 1 Cognition and Brain Plasticity Group [Bellvitge Biomedical Research Institute]- IDIBELL, L'Hospitalet de Llobregat, Barcelona 08907, Spain; 2 Epilepsy Unit, Neurological Service, Hospital Universitari de Bellvitge, L’Hospitalet de Llobregat, Barcelona 08907, Spain; 3 Department of Basic Psychology, Campus Bellvitge, University of Barcelona, L'Hospitalet de Llobregat, Barcelona 08907, Spain; 4 Catalan Institution for Research and Advanced Studies, ICREA, Barcelona, Spain; 5 Ecole Normale Superieure, Departement d’Etudes Cognitives, Paris, France

**Keywords:** Hippocampal sclerosis, Epilepsy surgery, Functional neuroimaging, Plasticity, Aphasia

## Abstract

**Background:**

Mesial temporal lobe epilepsy (MTLE) is the most common type of focal epilepsy in adults and can be successfully cured by surgery. One of the main complications of this surgery however is a decline in language abilities. The magnitude of this decline is related to the degree of language lateralization to the left hemisphere. Most fMRI paradigms used to determine language dominance in epileptic populations have used active language tasks. Sometimes, these paradigms are too complex and may result in patient underperformance. Only a few studies have used purely passive tasks, such as listening to standard speech.

**Methods:**

In the present study we characterized language lateralization in patients with MTLE using a rapid and passive semantic language task. We used functional magnetic resonance imaging (fMRI) to study 23 patients [12 with Left (LMTLE), 11 with Right mesial temporal lobe epilepsy (RMTLE)] and 19 healthy right-handed controls using a 6 minute long semantic task in which subjects passively listened to groups of sentences (SEN) and pseudo sentences (PSEN). A lateralization index (LI) was computed using a priori regions of interest of the temporal lobe.

**Results:**

The LI for the significant contrasts produced activations for all participants in both temporal lobes. 81.8% of RMTLE patients and 79% of healthy individuals had a bilateral language representation for this particular task. However, 50% of LMTLE patients presented an atypical right hemispheric dominance in the LI. More importantly, the degree of right lateralization in LMTLE patients was correlated with the age of epilepsy onset.

**Conclusions:**

The simple, rapid, non-collaboration dependent, passive task described in this study, produces a robust activation in the temporal lobe in both patients and controls and is capable of illustrating a pattern of atypical language organization for LMTLE patients. Furthermore, we observed that the atypical right-lateralization patterns in LMTLE patients was associated to earlier age at epilepsy onset. These results are in line with the idea that early onset of epileptic activity is associated to larger neuroplastic changes.

## Background

Temporal lobe epilepsy (TLE), the most common cause of intractable epilepsy in adults, can be successfully cured by surgery [1]. Partial removal of the anterior temporal lobe (ATL) remains the most common form of surgical treatment and is effective in 60-80% of patients [1-3]. A complication of surgery observed in 30-50% of patients after left ATL resection is a decline in language or verbal memory function [4-7]. The magnitude of decline is related to the degree of language lateralization to the left hemisphere and can be predicted using preoperative functional magnetic resonance imaging fMRI; [6-8].

A decrease of regional cerebral metabolism unilateral to the epileptogenic focus has been observed in patients with intractable TLE, usually in temporal and occasionally prefrontal regions [9]. Similarly, the reorganization of language may also affect different productive (located in frontal areas) and perceptive (located in temporal regions) language functions mainly in temporal rather than frontal areas [10,11]. Atypical functional lateralization for refractory epileptic patients during fMRI language tasks has been widely reported with either bilateral or right lateralized patterns of activation [12]. This is predominant in patients suffering from left mesial temporal lobe epilepsy LMTLE; [6,11,13-15].

Although the tasks employed by epilepsy institutes vary greatly, most fMRI paradigms used to determine the language dominance in epileptic populations include language production paradigms such as word or verb generation [11,13,15-18], object naming [14], or sentence repetition [11]. More complex paradigms involving semantic decisions have also been used [6,7,12,18,19]. All these paradigms require the patient to perform an active action (e.g., naming a word, making a semantic decision) which critically depends on his/her capacity to collaborate during the execution of the task. Paradigms that are too complex might result in patient underperformance, yielding poor activation patterns.

Surprisingly, only a few studies of epilepsy sufferers have used pure passive tasks such as listening to standard speech [11,20,21]. Passive listening paradigms reliably activate the receptive language cortex [22] making it possible to determine hemispheric dominance and identify the areas involved in language processing of TLE patients, which is of the utmost importance in pre-surgical language mapping [10,11]. These tasks commonly bring about activations in Wernicke's area –mainly the superior temporal gyrus (STG) and the middle temporal gyrus (MTG), frequently extending into the angular gyrus- but also in the primary auditory area and less often in frontal regions [11,20,23,24]. A study comprising healthy children [24] showed that both passive listening and active-response story processing yielded activations in the primary auditory cortex and the STG bilaterally and also in the left inferior frontal gyrus (IFG). It has also been shown in TLE patients with atypical language patterns that plastic cortical reorganization can affect frontal and temporal language areas differently with greater reorganization of language circuits in the temporal rather than the frontal lobe [11,20]. This suggests that productive and receptive functions can be affected by the pathological process in different ways. In contrast with productive tasks, passive tasks are easy to perform, less dependent on patient collaboration and allow the assessment of how fronto-temporal networks contribute to other aspects of language processing. In addition, the pattern of activation of some receptive language paradigms can be as lateralized as that of verbal fluency or semantic decision tasks [11,20,22-25]. However, it is also well-known that using a baseline condition such as fixation or rest can lead to more bilateral activations [26].

The main purpose of this study is to investigate the reliability of a passive, non-collaboration dependent, semantic fMRI language task in evaluating language lateralization patterns in a group of selected left and right mesial temporal lobe epilepsy (RMTLE) patients. These results were compared to those of a well-matched healthy group. Based on previous studies, we expected to observe a clear increase of right-hemisphere lateralization associated to earlier age at epilepsy onset in LMTLE patients [12,27].

## Methods

### Participants

Twenty-three native Spanish patients (15 women) with refractory epilepsy of the temporal lobe were evaluated using an fMRI passive language paradigm. Twelve patients had a left hemispheric focus (LMTLE group) with the remaining eleven exhibiting the right (RMTLE group). All patients underwent pre-surgical evaluation which included long-term in-patient video-electroencephalogram (video-EEG) monitoring with clinical and EEG assessment, neuropsychological testing, brain MRI and psychiatric evaluation. All patients were considered good candidates for resective mesial temporal lobe epilepsy (MTLE) surgery. Twenty-two patients were right handed with only one left-handed right MTLE patient, as assessed by the Edinburgh handedness test [28]. Structural MRI on a 3 T Siemens Trio MRI system showed mesial temporal sclerosis in all patients with no other lesions found.

Nineteen healthy native Spanish subjects (9 women) were studied using the same fMRI and neuropsychological protocols. All controls were right handed as assessed by the Edinburgh handedness test [28] and had no record of neurological illness. None of the patients or controls had hearing disabilities.

We found no statistical differences between groups (n.s, all p > 0.4, see Table 1) in age (RMTLE, LMTLE, controls) or age at epilepsy onset (RMTLE, LMTLE). The study was approved by the Ethical Committee of University Hospital of Bellvitge and informed consent was obtained from all patients and controls.

**Table 1:** Demographics, neuropsychological results and mean LI for each group and contrast

	**LMTLE (n = 12)**	**RMTLE (n = 11)**	**Controls (n = 19)**
Age	40.50 ± 10.11	44.81 ± 13.51	40.68 ± 14.69
Age at epilepsy onset	14.94 ± 14.68	15.96 ± 12.01	-
Years of education	11.08 ± 3.87	11.54 ± 2.91	14.52 ± 4.35
Edinburg Handedness Inventory	1.00 ± 0.00	1.45 ± 1.21	1.11 ± 0.32
IQ	92.50 ± 9.17	97.81 ± 11.19	106.35 ± 12.35
BNT (SS)	48.08 ± 6.15	51.27 ± 5.42	51.42 ± 8.80
Phonemic fluency (SS)*	13.08 ± 6.67	12.81 ± 6.17	15.21 ± 5.74
Semantic fluency (SS)*	18.08 ± 5.46	18.18 ± 4.19	19.78 ± 5.70
Immediate verbal memory (SS)**	30.08 ± 11.10	27.63 ± 6.12	32.68 ± 13.26
Delayed verbal memory (SS)**	14.91 ± 7.93	16.63 ± 5.57	20.42 ± 10.01
**Lateralization index**			
**LI: SEN vs. Rest**	**-0.32 ± 0.31**	**-0.10 ± 0.40**	**0.03 ± 0.34**
**LI: PSEN vs. Rest**	**-0.41 ± 0.27**	**-0.16 ± 0.29**	**-0.08 ± 0.32**

LMTLE, Left Temporal Lobe Epilepsy; RMTLE, Right Temporal Lobe Epilepsy; SS, scaled score; IQ, Intelligence Coefficient estimation; BNT, Boston Naming Test; *Subtests of the Barcelona test; **Subtest of the Wechsler Memory Scale III; SEN, Passive Listening to Sentences; PSEN, Passive Listening to Pseudo Sentences.

All values are reported with mean and standard deviation. LIs lower than -0.4 are considered right lateralized, with LIs larger than 0.4 being considered left lateralized. LIs between -0.4 and 0.4 are considered bilateral.; For the handedness the scores were evaluated according to the following scale 1 = Right and 5 = Left; (for the interpretation of the results see methods section).

### Neuropsychological testing

Neuropsychological data are summarized in Table 1. All patients and controls completed a set of subtests from the Wechsler Memory Scale III and Wechsler Adult Intelligence Scale III (immediate and delayed verbal memory; immediate and delayed visual memory) [29]. The Boston Naming Test [30] and Semantic and Phonological fluency tests [31] were also carried out in order to explore naming abilities and verbal fluency. All these scores were compared to normative data, minimizing the possible bias of age and education in further statistical analysis. No statistical differences between the RMTLE group, LMTLE group and controls were found in any of the neuropsychological tests (n.s., all *p* > 0.15); however a marginal effect was found for years of education (n.s., p > 0.06). Following previous studies [6] we ensured that all participants had an IQ estimation ≥70, measured by the Vocabulary subtest of the Wechsler Adult Intelligence Scale III.

### MRI scanning

Images were acquired using a 3.0 Tesla Siemens Trio MRI system at the Hospital Clinic of Barcelona. A T1-weighted image (slice thickness = 1 mm; no gap; number of slices = 240; TR = 2300 ms; TE = 3 ms; matrix = 256 × 256; FOV = 244 mm) was acquired. Two functional runs of 90 echo-planar images were acquired using a single-shot T2*-weighted gradient-echo EPI sequence (slice thickness = 4 mm; no gap; number of slices = 32, interleaved order; TR = 2000 ms; TE = 29 ms; flip angle = 80°; matrix = 128×128; FOV = 240 mm; voxel size = 3 × 3 × 4 mm^3^).

### Experimental design

Stimuli consisted of 20 well-formed Spanish sentences (SEN) containing 8 words (e.g., "*En el museo hay una exposición de mariposas*", meaning "*There is an exhibition of butterflies in the museum*") and 20 meaningless pseudo sentences (PSEN) (e.g., "*An er sureo tas uma emdoriciós ne sacidosar*"). PSENs were derived from well-formed sentences by substituting phonemes based on Spanish phonotactical rules, thus matching the original stimuli in phonemic complexity and length [32]. All stimuli were recorded using a native Spanish speaker reading the sentences. Patients were instructed to listen carefully to the stimuli as they would be required to answer some questions at the end of the session.

The stimuli was presented over two runs of a blocked design paradigm. Each run contained three experimental conditions: passive listening to SENs, passive listening to PSENs and rest. SEN and PSEN blocks contained 5 sentences each, with a total block duration of 30 seconds and were each followed by 15 second rest periods. Each run contained two blocks of SEN and PSEN conditions and 4 of rest, yielding a total task duration of 6 minutes over the two runs.

### fMRI analysis and Lateralization Index calculation

Analysis of fMRI data was carried out using Statistical Parametric Mapping software (SPM8; Wellcome Department of Imaging Neuroscience, University College, London, UK, http://www.fil.ion.ucl.ac.uk/spm) and MATLAB 7.8.0 (The MathWorks Inc, Natick, Mass). Echo-planar imaging (EPI) volumes were first spatially realigned and resliced with respect to the first volume of the first run. Each participant’s T1 image was coregistered to the mean EPI volume produced in the previous spatial realignment step. Then, using each coregistered T1 scan, the normalization parameters needed to transform the data to MNI152 space were obtained by means of Unified Segmentation [33], which combines segmentation, bias correction and spatial normalization under the same iterative model. This method has been successfully applied to both healthy and patient populations [34]. Finally, normalized EPI images were resliced to 2 × 2 × 2 mm and smoothed with an 8 mm full-width at half-maximum isotropic Gaussian kernel.

A general linear model was created using SEN, PSEN and rest conditions and the motion parameters extracted from the realignment phase. After model estimation, statistical parametrical maps for the SEN and PSEN conditions versus rest and for SEN versus PSEN were created. The first objective of this study was to assess whether a passive semantic task as the one presented could elicit robust activation of the temporal lobe at the subject-level. Thus, individual first level contrasts were inspected in order to compare the activations in individual subjects between active task and rest contrasts (SEN vs. rest, PSEN vs. rest) and contrasts involving only active blocks (SEN vs. PSEN) using a p < 0.001 uncorrected threshold with 20 voxels of cluster extent. The SEN vs. PSEN contrast did not elicit significant differences in around 40% of the subjects (see Results) and no laterality indexes or group contrasts were derived. No further analysis was conducted here as a contrast that shows no robust activations in all participants is of poor clinical use.

Active versus rest contrasts (PSEN vs. rest and SEN vs. rest) however elicited activations at the subject level in 100% of the participants (see Results) and were therefore both well-suited for clinical use. Group activations were then calculated using a one-sample t-test. These contrasts were calculated to provide a general idea of the pattern of activations in each group and therefore are reported at a false discovery rate corrected (FDR-corrected) p < 0.05 threshold with 20 voxels of cluster extent, to correct for multiple comparisons.

**Figure 1:**
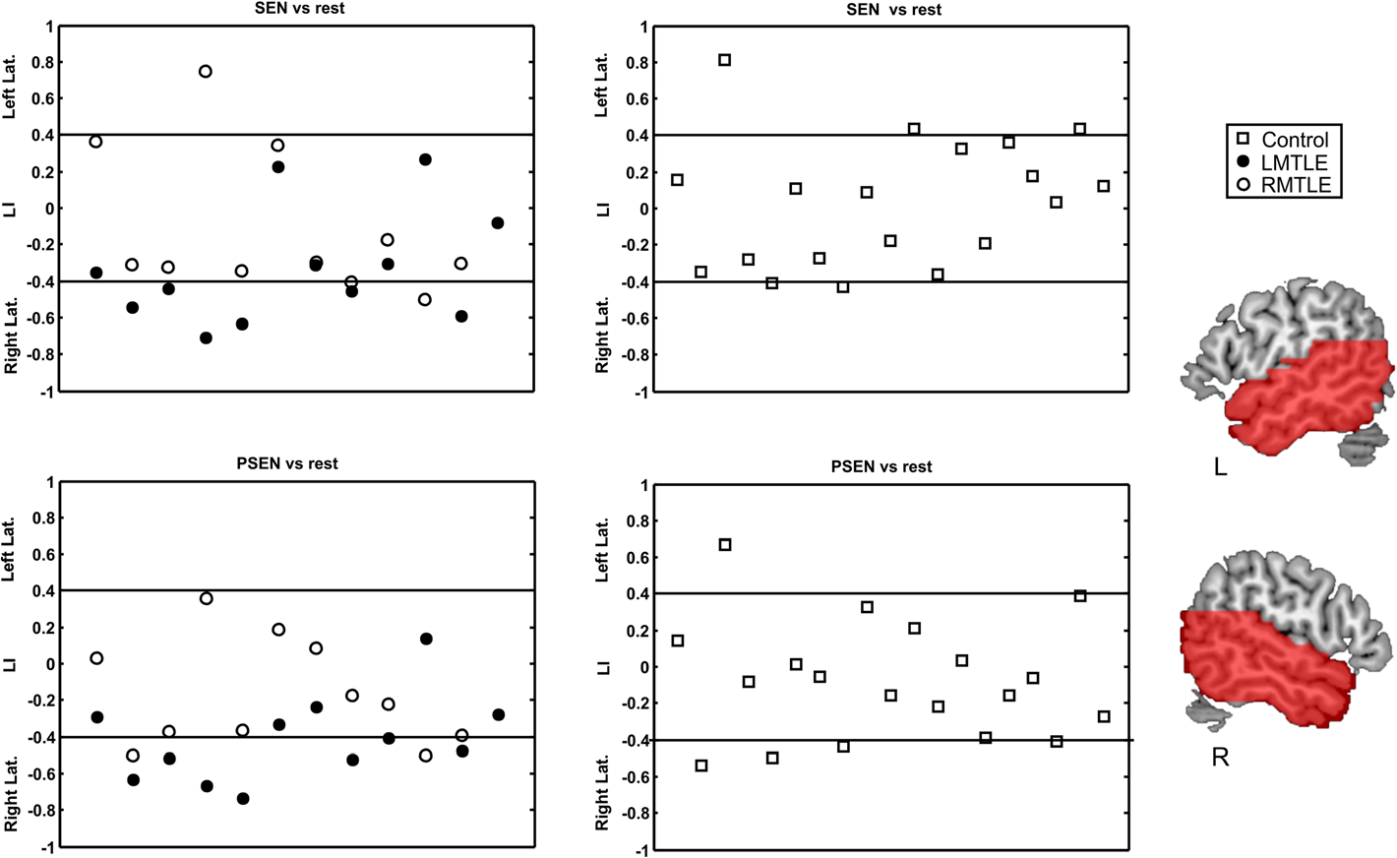
**Individual LIs for each participant and for both SEN vs. Rest and PSEN vs. Rest contrasts.** LIs under -0.4 are considered right lateralized, with LIs being larger than 0.4 being considered left lateralized. LIs between -0.4 and 0.4 are considered bilateral. On the right side the ROIs used to calculate the LIs are overlaid on red over a canonical MNI template. PSEN, Passive Listening to Pseudo Sentences; SEN, Passive Listening to Sentences; LMTLE, Left Temporal Lobe Epilepsy; RMTLE, Right Temporal Lobe Epilepsy; L, Left Hemisphere; R, Right Hemisphere.

For each subject and for both PSEN vs. rest and SEN vs. rest contrasts, a Lateralization Index (LI) was calculated using a region of interest (ROI) which included the STG, MTG, inferior temporal gyri (ITG) and the anterior temporal lobe [35]; see Figure 1. The rationale behind this anatomical selection is based on the importance of ATL regions in MTLE patients, areas which are typically resected for epilepsy treatment [10,11]. The ROIs were generated using the Automated Anatomical Atlas [36] and the toolbox WFU PickAtlas [37,38]. The LI is calculated as:

(1) $$\mathrm{LI}=\frac{\left(\sum \mathrm{LeftActivation}-\sum \mathrm{RightActivation}\right)}{\left(\sum \mathrm{LeftActivation}+\sum \mathrm{RightActivation}\right)}$$

Thus, the LI ranges between 1 for extreme left lateralized activations and -1 for total right lateralization patterns. In other words, an LI greater than 0.4 means that at least 70% (following eq. 1, 0.4 = [30]/100) of activation is left lateralized, while a LI below -0.4 implies that at least 70% of activation (-0.4 = [30-70]/100) is in the right hemisphere. This criterion was the threshold selected to classify a pattern of activation as left or right lateralized with LIs between 0.4 and -0.4 being considered bilateral. LIs were derived using the bootstrapped method (in which no threshold needs to be specified as this particular algorithm is threshold-independent) of the LI-toolbox [39], in which 10.000 LIs per subject were iteratively calculated at different thresholds using voxel values and clustering, yielding a robust LI.

A one-way ANOVA on LIs was performed to check for a significant group effect (RMTLE, LMTLE, controls). Two sample *t*-tests were also calculated between pairs of groups to evaluate significant differences in their mean LI. LIs were also correlated with the age at epilepsy onset and all neuropsychological variables (for each group and pooling all the groups together) using Pearson's linear correlation. For significant correlations and trends, 95% bootstrapped confidence intervals (CI) were calculated using 10.000 permutations. In this manner, if CIs are greater than zero, the probability of the correlation being non significant is reduced to 5%.

## Results

### fMRI results

**Figure 2:**
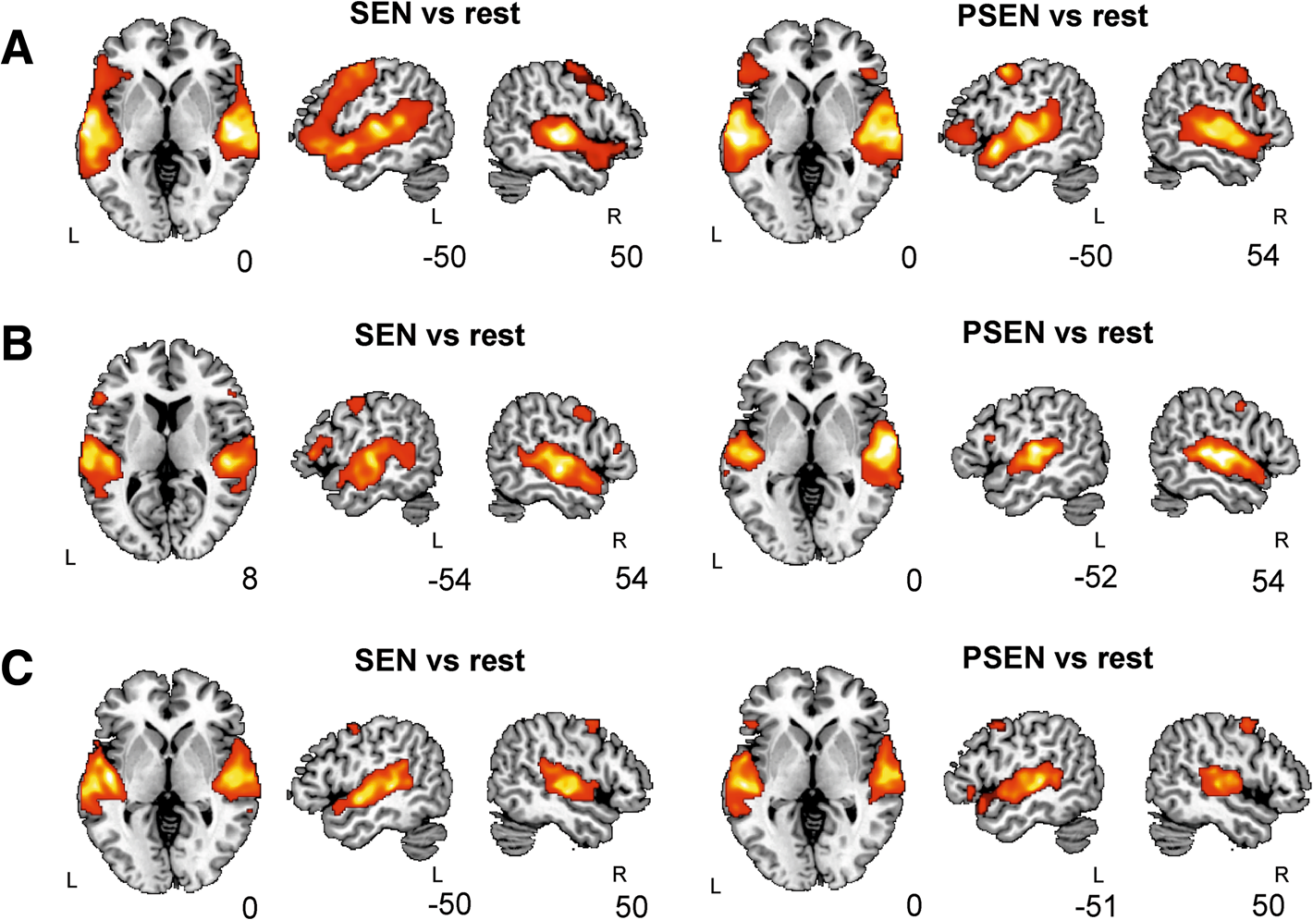
**Whole brain fMRI activations for the three groups.** Results are shown over an MNI template, at a *p* < 0.05 FDR-corrected threshold with 20 voxels of cluster extent using neurological convention. **A**. Healthy participants **B**. LMTLE patients. **C**. RMTLE patients. PSEN, Passive Listening to Pseudo Sentences; SEN, Passive Listening to Sentences; L, Left hemisphere; R, Right hemisphere.

**Figure 3:**
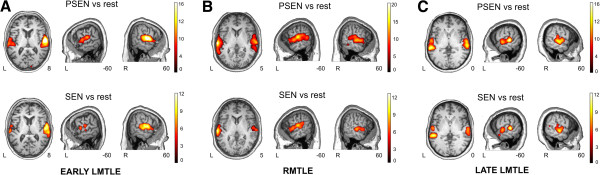
**Whole brain fMRI activations for three representative patients.** Results are shown over a patient's T1, at a *p* < 0.05 FDR-corrected threshold with 20 voxels of cluster extent using neurological convention. **A**. Patient 9 from the LMTLE group (woman, 63 years old, age at epilepsy onset at 3 years, LI for PSEN -0.65, LI for SEN -0.77). **B**. Patient 14 from the RMTLE group (man, 23 years old, age at epilepsy onset at 0.66 years, LI for PSEN 0.19, LI for SEN 0.35). **C**. Patient 27 from the LMTLE group (woman, 48 years old, age at epilepsy onset at 32 years, LI for PSEN 0.14, LI for SEN 0.27). PSEN, Passive Listening to Pseudo-sentences; SEN, Passive Listening to Sentences; L, Left hemisphere; R, Right hemisphere.

Task vs. rest contrasts (SEN vs. rest, PSEN vs. rest) thresholded at *p* < 0.001 uncorrected with 20 voxels of cluster extent and elicited clear activations in the STG and MTG bilaterally in all patients and controls (42 subjects in total, see Figure 2 and Tables 2, 3 and 4 for descriptions of each contrast of interest per group and Figure 3 for single case examples). The contrast between SEN vs. PSEN failed to show significant activations in any cortical region in 4 out of 11 (36%) RMTLE patients, 5 out of 12 (42%) LMTLE patients and in 8 out of 19 (42%) controls and thus was not analyzed any further.

**Table 2:** Main fMRI results on the functional tasks for the control group

	**Side**	**Cluster size (mm3)**	***t *** **value**	**Peak coordinate**		
				**x**	**y**	**z**
**Healthy population**						
SEN vs. rest						
STG/MTG/HG/IFG	R	40112	16.88	56	-20	0
STG/MTG/HG/IFG/PG	L	81928	14.64	-62	-16	-6
SMA	L/R	7096	8.85	-4	4	66
PG	R	5560	5.30	56	12	30
PSEN vs. rest						
STG/MTG/HG/	L	46552	16.47	-58	-24	2
STG/MTG/HG/	R	45952	13.47	42	-20	6
PG/PC	L	3264	9.04	-50	-2	52
IFG	L	4936	5.47	-54	30	0
IFG	R	1264	5.27	60	22	20
PG	R	1328	4.04	54	0	46

All peak values reported were significant at *p* < 0.05 FDR-corrected at voxel level, 20 voxels spatial extent. Peak coordinates of each cluster are reported in MNI coordinates. STG, Superior Temporal Gyrus; MTG, Middle Temporal Gyrus; HG, Heschl Gyrus; PC, Postcentral Gyrus; PG, Precentral Gyrus; SMA, Supplementary Motor Area; IFG, Inferior Frontal Gyrus.PSEN, Passive Listening to Pseudo Sentences; SEN, Passive Listening to Sentences; L, Left hemisphere; R, Right hemisphere.

**Table 3:** Main fMRI results on the functional tasks for RMTLE group

	**Side**	**Cluster size (mm3)**	**t value**	**Peak coordinate**		
				**x**	**y**	**z**
**RMTLE**						
SEN vs. rest						
STG/MTG/HG	R	21912	12.83	60	-8	8
STG/MTG/HG/	L	27280	12.49	-54	-28	4
PG	R	6528	7.42	52	2	54
PG	L	712	5.18	-50	8	52
IFG	L	432	4.78	-46	26	2
SMA	L/R	424	4.74	4	6	60
PSEN vs. rest						
STG/MTG/HG	L	33216	13.92	-54	-30	6
STG/MTG/HG/	R	28256	11.89	62	-24	6
PG	R	584	5.76	54	6	48
SMA	L/R	256	5.11	4	0	68
PG	L	248	4.88	-54	-2	50

All peak values reported were significant at p < 0.05 FDR-corrected at voxel level, 20 voxels spatial extent. Peak coordinates of each cluster are reported in MNI coordinates. STG, Superior Temporal Gyrus; MTG, Middle Temporal Gyrus; HG, Heschl Gyrus; PG, Precentral Gyrus; SMA, Supplementary Motor Area; IFG, Inferior Frontal Gyrus. PSEN, Passive Listening to Pseudo Sentences; SEN, Passive Listening to Sentences; L, Left hemisphere; R, Right hemisphere.

**Table 4:** Main fMRI results on the functional tasks for LMTLE group

	**Side**	**Cluster size (mm3)**	**t value**	**Peak coordinate**		
				**x**	**y**	**z**
**LMTLE**						
SEN vs. rest						
STG/MTG/HG	R	28952	14.90	62	-18	4
STG/MTG/HG/	L	26344	12.71	-64	-28	6
PG/PC	L	1832	7.70	-46	-10	56
IFG	L	1504	7.13	-54	28	8
PG	R	1424	5.36	60	0	40
IFG	R	208	4.75	52	30	12
SMA	L/R	424	4.56	2	0	64
PSEN vs. rest						
STG/MTG/HG	R	29064	23.58	58	-16	4
STG/MTG/HG/	L	21416	16.56	-62	-28	10
PG	R	480	5.42	56	-2	42
IFG	L	424	4.49	-52	16	18

All peak values reported were significant at p < 0.05 FDR-corrected at voxel level, 20 voxels spatial extent. Peak coordinates of each cluster are reported in MNI coordinates. STG, Superior Temporal Gyrus; MTG, Middle Temporal Gyrus; HG, Heschl Gyrus; Postcentral Gyrus; PG, Precentral Gyrus; SMA, Supplementary Motor Area; IFG, Inferior Frontal Gyrus. PSEN, Passive Listening to Pseudo Sentences; SEN, Passive Listening to Sentences; L, Left hemisphere; R, Right hemisphere.

### Lateralization index

The mean LI and standard deviation for each group and condition can be found in Table 4, while individual LIs are shown for every participant and both SEN vs. rest and PSEN vs. rest contrasts in Figure 1.

For the SEN vs. rest contrast, a one-way ANOVA analysis on LIs showed a significant group effect (*F*(2,39) = 3.69 *p* < 0.035). The predominant pattern of activations was bilateral for controls (mean LI 0.03, 3 subjects left lateralized, 1 right dominant and 15 bilateral) and RMTLE patients (mean LI -0.10, 1 patient left lateralized, 1 right lateralized and 9 bilateral). LMTLE patients (6 patients with right hemispheric dominance and 6 bilateral) showed the most right lateralized pattern of activation (mean LI -0.32, 65% of activation in right temporal lobe) which was significantly more right lateralized than controls (*t*(29) = -2.87, *p* < 0.008). No significant differences were found between the mean LI of LMTLE and RMTLE patients (*t*(21) = -1.44, *p* > 0.16) or RMTLE patients and controls (*t*(28) = -0.98, p > 0.33). Correlations between LIs for this contrast and age at epilepsy onset for the RMTLE patients were not significant (*r* = -0.15, *p* > 0.65). However, for the LMTLE group a small trend was observed (*r* =0.40, *p* > 0.19, bootstrapped CIs -0.20/0.88). No other significant correlations or marginal effects were found (all p > 0.20).

**Figure 4:**
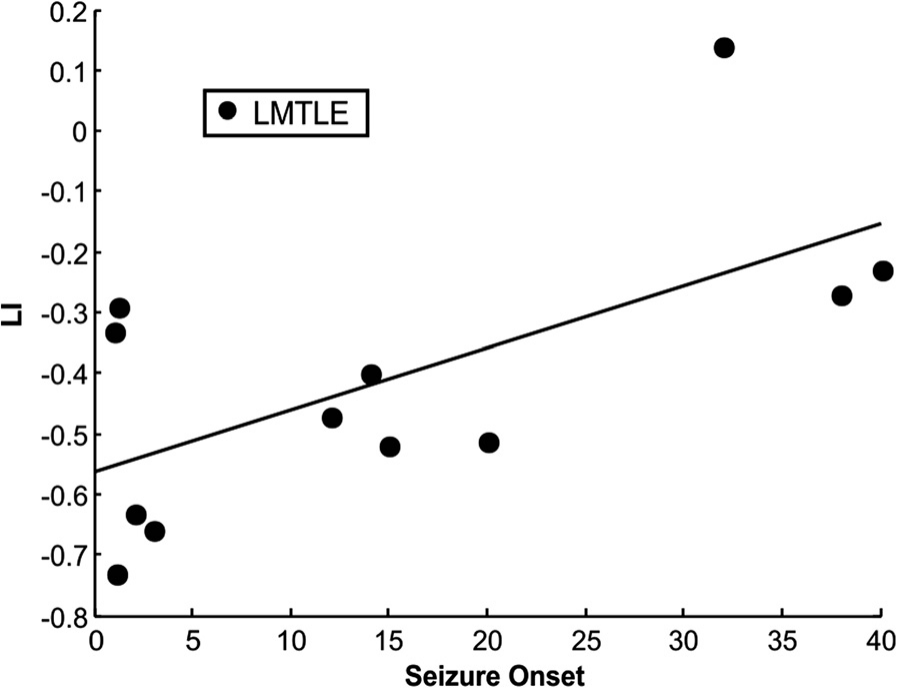
On the left side, LIs correlation for the PSEN vs. rest of LMTLE patients and their epilepsy onset age (r = 0.63, p < 0.03).

One-way ANOVA analysis on LIs in the PSEN vs. rest contrast also showed a significant group effect (*F*(2,39) = 4.67 *p* < 0.015). For controls (mean LI -0.08, 1 subject with left hemispheric dominance, 3 right lateralized and 15 bilateral) and RMTLE patients (mean LI -0.16, 2 patients right lateralized and 9 bilateral) bilateral activation was commonly observed. LMTLE patients (6 patients with right hemispheric dominance and 6 bilateral) showed, once again, the most right lateralized pattern of activation (mean LI -0.41, 70% of the activation in the right temporal lobe) which was significantly more right lateralized than controls (*t*(29) = -3.03, *p* < 0.005) and RMTLE patients (*t*(21) = -2.18, *p* < 0.041). No significant differences were found between the mean LI of RMTLE patients and controls (*t*(28) = -0.73, *p* > 0.47). The correlation between LIs for the PSEN contrast and age at epilepsy onset for RMTLE patients was not significant (r = -0.1, *p* > 0.76), whereas for the LMTLE group a significant correlation was found (see Figure 4) (*r* = 0.63, *p* < 0.03, bootstrapped CIs 0.16/0.93). No other significant correlations were found (all p > 0.20).

### Individual cases of interest

In order to illustrate the results, Figure 3 shows the main activations for SEN vs. rest and PSEN vs. rest contrasts for three representative patients. To emphasize the strength of the activations found in the temporal lobe at the subject-level, an FDR-corrected *p* < 0.05 threshold with 20 voxels of spatial extent was used to show the activations in these particular three subjects. Figure 3A shows the results of Patient 9, a 63 year old woman suffering from LMTLE with an early epilepsy debut (3 years), showing a strongly right lateralized activation pattern in both PSEN (LI -0.65, 82.5% of activation in the right temporal lobe) and SEN conditions (LI -0.77, 88.5% of activation in the right temporal lobe). Figure 3B shows the results of Patient 14, a 23 year old man suffering RMTLE with early epilepsy debut (0.66 years), showing bilateral (slightly left lateralized) activation in both PSEN (LI 0.19, 59.5% of activation in the left temporal lobe) and SEN conditions (LI 0.35, 67.5% of activation in the left temporal lobe). Figure 3C shows the results of Patient 27, a 48 year old woman suffering LMTLE with late epilepsy debut (32 years), showing bilateral activation in both PSEN (LI 0.14, 57% of activation in the left temporal lobe) and SEN conditions (LI 0.27, 63.5% of activation in the left temporal lobe).

## Discussion

In line with previous intracarotid amobarbital procedure data and consistent with our findings, several fMRI studies have suggested that atypical dominance patterns are more frequent in epileptic patients [12,17,19,40] and may predominate in patients with epilepsy of left hemispheric origin [13,19,41]. A previous study [41] showed that 24% of LMTLE patients with HS had atypical language dominance and more importantly that this atypical language representation was associated with both a higher frequency of interictal discharges and with sensory auras representing seizure propagation to lateral temporal structures. It has been suggested that propagation of seizures from the epileptic focus to different brain regions triggers cortical plasticity not only around the focus but also in remote areas either ipsilateral or contralateral to the epileptic focus [13,14,17,19,40]. It would therefore appear that the language network is particularly vulnerable to chronic epileptic activity. Moreover, an inter-hemispheric dissociation of frontal and temporal language regions in patients with focal epilepsy has also been described [10,11,20,42-44]. In a previous study, 29 (20.1%) out of 144 patients with medically intractable complex-partial seizures showed bilateral language representation after intracarotid amobarbital test assessment [10] and more importantly, 4 (2.8%) of these patients -2 of them with TLE- had strong evidence of an interhemispheric dissociation of expressive and receptive language functions. These findings have been posteriorly confirmed by several functional neuroimaging studies [11,20,42-44], which all support the hypothesis that reorganization can affect frontal and temporal language areas differently in patients with atypical language patterns.

Paradigms used for clinical language mapping vary greatly in different centres and no accepted standardized technique exists. Most fMRI language dominance studies in epileptic patients have used active language tasks [13-18]. However, imaging studies have confirmed that the laterality indexes yielded by receptive language paradigms can be as lateralized as those of active tasks [11,20,22-25]. In this way, and similarly to other passive listening semantic paradigms used on TLE patients [11,20,21], we show how passive contrasts such as listening to SENs or PSENs versus baseline are able to unravel and clarify atypical language patterns in LMTLE patients. Mean LI was significantly more right lateralized for LMTLE patients than controls when listening to SENs and again more right lateralized for LMTLE than for both controls and RMTLE patients when listening to PSENs (see Figure 1 and Table 1). While LI was mainly bilateral for the controls (79% of subjects for both SEN and PSEN conditions) and for RMTLE patients (81.8% of patients, for both SEN vs. rest and PSEN vs. rest conditions), 6 out of 12 patients of the LMTLE group showed a right lateralized activation for both contrasts (50% of patients).

It is important to note that this passive listening task is very easy and rapid to perform (only 6 minutes of fMRI acquisition) and that both task-rest contrasts produced robust activations of the temporal lobe for every one of the 42 participants studied. Interestingly, the SEN vs. PSEN contrast failed to elicit robust responses in 42% of LMTLE patients and controls and in 36% of RMTLE patients. These results are consistent with other passive paradigms using direct speech contrast between two active conditions, which also failed to show significant activations in several subjects. In a previous study [11], a direct contrast between two active conditions (Listening Stories vs. Listening Reversed Stories) produced no significant activation in 22% of RMTLE and LMTLE patients and 12% of healthy subjects. Interestingly, in another study [35], activation in 100% of subjects failed to reach significance in the direct contrast between two active conditions (listening to Words, Pseudowords and Reversed Speech).

In accordance with the present results, there is extensive functional imaging data suggesting that language comprehension is subserved by a large cortical network involving both temporal cortices [45-52]. Many neuroimaging studies reveal weak neural activity in the right hemisphere in the anatomically equivalent areas to those of the left hemisphere which show a strong signal during language tasks [48,51]. Additionally, some patients with right brain damage have deficits in comprehending natural language [48]. All these data support the idea that although the left hemisphere plays a crucial role in language processing, the right hemisphere also contributes to language comprehension [45,48,50,51,60]. Hence, the bilateral pattern of temporal lobe activation in controls and MTLE patients using this passive semantic paradigm agrees with previous functional imaging data showing that the STG and MTG are activated bilaterally by both speech and complex non-speech sounds [20,32,35,45,53-61]. Even when the auditory stimuli is novel and no meaning extraction is possible (as in the PSEN condition) both the MTG and STG show enhanced activations [62,63]. In fact, some studies have shown that activation at the level of the auditory cortex is neither modulated by the semantic content ("meaningfulness") of stimuli nor by the type of cognitive task performed [64]. Moreover, in a previous study [35] Words, Pseudowords and Reversed Speech versus baseline bilaterally activated both temporal lobes, yielding less than 2 ml of volumetric difference in activation between hemispheres (the only contrast that showed a significant difference in activation in temporal lobes was Words vs. rest with an LI of 0.11, therefore showing a bilateral distribution). It is also well known that that using a baseline condition such as fixation or rest (as in the SEN vs. rest and PSEN vs. rest contrast) yields bilateral functional patterns and LI values around zero [26]. There is also experimental and clinical evidence showing bilateral activation in conceptual processing [65].

Furthermore, a significant correlation between LI and age at epilepsy onset was found for the PSEN condition versus baseline (LIs for SEN vs. rest contrast also yielded a trend). There is a gradual decline in the potential for plasticity with age [12,27], but no particular age after which plasticity becomes absolutely impossible [66]. Visual inspection of patients with LMTLE and early onset clearly showed a right lateralized pattern (Figure 3A), while subjects suffering from RMTLE or LMTLE with late epilepsy debut showed a more bilateral pattern (Figure 4B and 4C, respectively). It is important to note that a correlation between age at epilepsy onset and LI has been previously described using a semantic decision task [12]. This gives additional support to the usefulness of a simple passive language task like the one described in this study.

Previous studies using language fMRI with productive and receptive tasks have shown how in TLE patients with atypical language patterns, reorganization affected frontal and temporal language areas differently, with temporal LIs being more right lateralized than frontal ones [10,11,20,42-44]. Moreover, data from lesion and neuroimaging studies suggests that comprehension or semantic activation -involving retrieval and selection of semantic information- depends on a wide cortical network, larger than Wernicke's or even Broca’s area (which is required particularly for the correct processing of complex morphosyntactic structures) [67]. Furthermore, there are several patient studies supporting the involvement of the anterior superior temporal lobe for sentence-level comprehension [45,65,67,68]. Semantic knowledge stores are diffusely located in ventral temporal (MTG, ITG, fusiform gyrus, temporal pole) and inferior parietal (angular gyrus) cortices, in addition to the classic posterior STG and planum temporale [23,45,48,50,69,70]. Therefore, as both the ventral and anterior temporal lobes are related to receptive language and TLE surgery (ATL) commonly involves the resection of these regions of the temporal lobe, we hypothesize that the passive semantic paradigm presented here -which mainly activates temporal lobe structures- is more precise in predicting semantic language defects, such as naming retrieval or verbal memory decline, in TLE patients who undergo surgery [4,23,70-76]. In addition, this paradigm may also provide important information about the extent of functional reserves in the contralateral hemisphere [20] and might be helpful in tailoring the resection of temporal lesions with the aim of preserving eloquent areas of the brain. We cannot omit however that a limitation of this study is the lack of comparison to other laterality measurements yielded by more classical non-passive language paradigms. Also, a larger sample of patients might have allowed us to compute a more detailed analysis of the LIs within the temporal lobes by dividing the MTL into different sections (i.e., anterior/posterior).

## Conclusions

In summary, the simple, rapid, non-collaboration dependent, passive task described in this study produces a robust activation in the temporal lobe in both TLE patients and controls and illustrates a pattern of atypical language organization specifically in LMTLE patients.

### Financial disclosure statement and acknowledgments

This research has been supported by a predoctoral grant from the Spanish Government (FPU program AP2010-4179) to P.R., a predoctoral grant from Generalitat de Catalunya to D.L-B. (2010FI B1 00169), a predoctoral grant from the Bellvitge Biomedical Research Institute (program 06/IDB-001) to A.V.B., a Ramon y Cajal research program grant awarded to J.M.P. (RYC-2007-01614), Spanish Government grants (MICINN, PSI2011-29219 to A.R.F., PSI2009-09101 to J.M.P. and PSI2008-03885 to R.D.B.) and a grant from the Catalan Government (2009 SGR 93).We would like to thank all patients and healthy volunteers for their participation.

## Competing interests

None of the authors has any conflict of interest to disclose.

## Authors’ contributions

Conception and design (PR, JM, MF, ARF, JMP, RdDB); acquisition of data (JM, PR, MF, AV, DLB, MJ); drafting of the manuscript (PR, JM, ARF); analysis and interpretation of data (PR, JM); critical revision of the manuscript for important intellectual content (ARF, JMP, AV, PR, RdDB, DLB, MJ, MF, JM); statistical expertise (PR, ARF, JMP); administrative, technical, or material support (RdDB, JMP, PR, JM); study supervision (MF, ARF). All authors red and approved the final manuscript.

## Pre-publication history

The pre-publication history for this paper can be accessed here:

http://www.biomedcentral.com/1471-2377/14/98/prepub
